# Inhibited Intentionality: On Possible Self-Understanding in Cases of Weak Agency

**DOI:** 10.3389/fpsyg.2020.558709

**Published:** 2020-09-25

**Authors:** Line Ryberg Ingerslev

**Affiliations:** Department of Philosophy, Faculty of Humanities, Julius-Maximilians-Universität Würzburg, Würzburg, Germany

**Keywords:** unreflective actions, habits, consciousness, action, responsibility, diachronicity

## Abstract

The paper addresses the question of how to approach consciousness in unreflective actions. Unreflective actions differ from reflective, conscious actions in that the intentional description under which the agent knows what she is doing is not available or present to the agent at the moment of acting. Yet, unreflective actions belong to the field in which an agent experiences herself as capable of acting. Some unreflective actions, however, narrow this field and can be characterized by intentionality being inhibited. By studying inhibited intentionality in unreflective actions, the aim of the paper is to show how weaker forms of action urge us to expand our overall understanding of action. If we expand the field of actions such that it encompasses also some of the involuntary aspects of action, we are able to understand how unreflective actions can remain actions and do not fall under the scope of automatic behavior. With the notion of weak agency, the paper thus addresses one aspect of unreflective action, namely, “inhibited intentionality” in which an agent feels a diminished sense of authorship in relation to her possibility for self-understanding. The notion of weak agency clarifies how agency itself remains intact but can involve a process of appropriation of one’s actions as one’s own. With a diachronic account of consciousness in unreflective action, the paper accounts for possible self-understanding in cases where none seems available at the moment of action.

## Introduction

At any moment in any man’s waking and conscious life there is always a set of possible true answers to the questions—“What is he doing now?” For human beings, to be conscious is to have active intentions. (Hampshire, 1970, p. 169).

Which behavior deserves the status of an action or what characterizes human action is, and has been, widely debated. Many of our actions are carried out in an unthinking, unreflective way. The way we get out of bed in the morning, the way we drive to work, how we greet our colleagues; our routines and daily doings often go by without us noticing what we are doing. How do we describe consciousness in unreflective actions, and can such forms of behavior be described as actions at all? According to standard accounts in the philosophical theory of action (Bratman, 1987; Anscombe, 2001; Davidson, 2001), what constitutes an action is that it is done for a reason and that the agent knows the description under which his action is intentional. Thus, if I, unreflectively and inattentively, put my left shoe on my right foot, this mistake does not fall under the scope of an action. The action in this case is that I put my shoes on in order to get dressed, and I am aware of dressing more or less attentively. The left shoe on my right foot is an accident; it falls outside the scope of what I am conscious of when acting.^1^

Thus, if we follow the standard theory when we consider consciousness in action, we typically want to hold on to the following assumptions, as they are both intuitively plausible:

(1) My behavior deserves the status of action, when I am conscious of doing it for a reason under some description.
(2) The behavior for which I ought to feel responsible is the behavior that deserves the status of actions.

However, if we hold on to both assumptions, some cases of unreflective action pose a problem for the standard theory of action. The problem I want to draw attention to is that the scope of behavior that I am conscious of intentionally doing under some description is smaller than the scope of the behavior for which I intuitively feel responsible. This discrepancy between what I do and what I can feel responsible for is central to many of our daily routines; therefore, it deserves theoretical attention.

The following example illustrates the discrepancy in question: Every morning I greet my colleagues upon entering our shared office; one I greet formally; another I greet in a playful tone of voice. If I was asked why that is, or if my serious colleague asked me whether there is a reason, why I treat him less playfully, I would have no good answer. Yet, intuitively, I do feel responsible for treating him as less playful or the other one as less serious, for that matter, even though I had no intention to do so. Despite the fact that I greet my colleagues for a reason, and that my action is intentional under this description, I still intuitively feel responsible for aspects of my behavior of which I am not consciously aware. I am not aware of greeting them differently, and thus, part of my behavior is something I intuitively feel responsible for despite the fact that I am acting unreflectively but for a reason under some other description.

By contrast, let us say a third colleague is in the room and I do not greet this person despite having seen her. In this case, I am responsible for acting rudely by not greeting her. Or, if I close my eyes as a reflex because of the sharp sunlight coming in from the windows, I might put my hand in front of my eyes and wave it a little. However, this is not a greeting gesture, it is a reflex. Mere behavior of this kind is without communicative intent.

According to assumption 2, action and responsibility are coextensive. If I am responsible for something, it is because it is an action of mine. According to assumption 1, action and consciousness are coextensive. That is, if something is an action of mine, I am conscious of what I am doing under its intentional description. However, according to my example, there are certain ways of doing things of which I am not consciously aware but for which I do remain responsible. To phrase it differently: In the light of habits, routines, and other aspects of my doings that are not in the foreground of my conscious awareness, standard philosophical accounts of action face the challenge that there appear to be actions of mine that I am not consciously aware of as intentional under a description but for which I remain responsible.

When responding to this challenge from within the framework established by action theory, three logically possible strategies present themselves.

(1) We can keep assumptions 1 and 2 but deny the case: We can deny that the way I greet people differently can be part of the action for which I am responsible. I greet many people differently, but what I am responsible for is that I greet them, not how I do so, unless of course this is part of my reason for doing it. I greet a former partner differently than I greet my best friend and purposefully so. However, I find it compelling that there are indeed cases as the one described above and many others, where it is important to retain responsibility despite the lack of an intentional description under which I am conscious of the action as reasonable.
(2) Another logically possible strategy consists in keeping responsibility and action coextensive, even when I am not conscious of my action. In this case, we would keep assumption 2. Hence, there will be actions of which I am not conscious in the sense described above but for which I do remain responsible. This response argues that no strong form of reflective consciousness is required for the constitution of an action. Greeting my colleagues differently is thus an action of mine for which I am responsible, despite the lack of a consciousness of a reason for doing it. Thus, we deny assumption 1 but hold that even in cases of unreflective doings, I remain responsible for my behavior. In this way, the scope of what I am consciously aware of is smaller than the behavior for which I am responsible.
(3) A third logically possible strategy consists in wanting to keep action and consciousness coextensive. My actions are coextensive with my behavior where I am conscious of the description that makes it intentional. The unreflective manner in which I greet someone is thus not part of my action. We keep assumption 1. This means that the field of actions is smaller than the behavior for which I am responsible: Unreflective doings fall outside the scope of action, but we might still be responsible for them. We thus deny assumption 2 and acknowledge that I can be responsible even for forms of unreflective behavior and not only for conscious actions.
  Both the second and third strategy take the greeting example seriously, but they do so at the expense of one of the intuitive assumptions we began with. I wish to propose a fourth strategy.
(4) I will argue that we can keep both assumptions but that they must be interpreted diachronically. In greeting my colleagues differently in an unthinking manner, I am unaware of doing so, but I do remain responsible for what I do. At the time of my action, I am not aware of the description that makes my action intentional, but over time, I can come to appropriate my behavior as an action of mine by subsequently becoming aware of a description under which it was indeed intentional. Thereby, I retrospectively take responsibility for it. As long as the description under which my earlier doing was intentional can become accessible to me at a later point, this doing will be an action for which I am responsible. I thus distinguish between intention in action and the appropriation of an intention of an earlier action. The difference will be explained in the following sections.

## Synchronicity

In what follows, I will look more closely at the first three logically possible strategies while arguing that a synchronicity assumption is a stake in them, that is, that the necessity of synchronicity of action consciousness and the action itself is assumed. In the case, where we keep both assumptions 1 and 2, one must deny that my manner of greeting—unless it is part of the description under which my action is intentional—can be part of my action. The agent must know the description under which an action is intentional: I am going to work (independently of whether I am attentive to the route); I am thinking about a job interview (independently of whether I bite my nails). Importantly, an intention cannot be ascribed *post hoc*; it has to be involved as a reason for one’s action. Thus, nail biting and the route I follow are mere happenings. Habitual actions that are characterized by such involuntary aspects fall outside the scope of what characterizes us as agents, of what constitutes our practical identities, and ultimately of what we are responsible for as agents. The habit of talking to myself while typing is an activity that is not an intentional action of mine (Setiya, 2017). Without the possibility of first-personal avowal or the acknowledgment of authority of my doings (Moran, 2001), they fail to be actions of the kind that constitutes my practical identity. In such cases, I will typically have merely attributional knowledge of my state of mind, based on observation, and mediated by some identifying description.^2^ In some cases, I might be immediately aware of my behavior yet lack the possibility of avowing what I am doing. However, in such cases, I would feel self-alienated rather than aware of myself as an agent (ibid., p. 33).

The problem with this strategy is that we are forced to narrow the field of action too far beyond our intuitive understanding of what counts as behavior for which I am responsible. To avoid this, some have suggested that we loosen the connection between reflective consciousness and action, as described in option (2) above. They have argued that we should still ascribe the status of actions to some of the behavior that we are not conscious of doing for a reason (Hursthouse, 1991; Steward, 2009; Owens, 2017). On this view, many of our actions we do unreflectively, but still, they remain actions.

A similar strategy has been pursued in the phenomenological tradition, where the link between consciousness and action is maintained by weakening the notion of consciousness required for behavior to count as action. The notion of embodied, operative intentionality serves to bring behavior within the scope of consciousness without requiring the stronger form of consciousness of intention under a description that is typically pursued in the analytical tradition. In my practical engagement in dancing, hammering, and walking, I embody intentionality, rather than reflectively carrying out a conscious action plan. Or, to put it differently, consciousness is broadened such that it not only refers to reflective consciousness in the sense that I know the description under which my action is intentional or done for a reason. Rather, it also includes forms of unreflective, embedded, and enactive consciousness. According to Merleau-Ponty, for instance,

Consciousness is originarily not an “I think that,” but rather an “I can” [. . . ] Consciousness is being toward the thing through the intermediary of the body. A movement is learned when the body has understood it, that is, when it has incorporated it into a subject’s “world,” and to move one’s body is to aim at the things through it, or to allow one’s body to respond to their solicitation, which is exerted upon the body without any representation. (Merleau-Ponty, 2012, pp.139–140).

As Merleau-Ponty writes: “My body has its world, or understands its world without having to go through ‘representations,’ or without being subordinated to a ‘symbolic’ or ‘objectifying function”’ (ibid., p. 141). This means that when the body acquires a habit, it comes to understand something in the world, and this understanding is practical: “This formula will seem absurd if ‘understanding’ is the act of subsuming a sensory given under an idea, and if the body is a mere object.” (ibid., p.146). Merleau-Ponty’s notion of habit clearly differs from any notion that would reduce habit to an automatic reflex, a tic or an otherwise involuntary side effect. Rather, habits are a form of practical, embodied understanding: “To understand is to experience the accord between what we aim at and what is given, between the intention and the realization—and the body is our anchorage in a world” (ibid., p.146). The affordance character of objects, situations, and other people (ibid., pp. 191–192) is perceived through the lived body, and the responsive answering of these calls is experienced as an embodied capacity. The “I can” is an experiential structure that shapes our bodily existence. Understanding is an embodied practice that precedes theoretical knowledge. In this way, embodied intentionality is operative beneath our consciously minded actions. Employing a broader conception of consciousness in action in this particular manner results in non-reflective embodied engagement figuring as a form of action. In such a theory, the domain of action is bigger than the domain of reflective, conscious doings. The focus is on the agent’s engagement and on embodied intentionality; hence, in terms of a theory of action, this means expanding the field of action such that even unreflective forms of behavior count as actions.^3^ Within analytical philosophy, we find similar arguments that reflective consciousness is not necessary for behavior to count as action. As Steward (2009) argues, it is the first personal capacity to do otherwise, which settles whether something is an action or not. When doing something unreflectively such as fiddling with one’s jewelry, it is one’s capacity to do otherwise that settles whether it is an action. She thinks that we should not overmentalize what constitutes an action. Rather, the two-way force consists in the ability of doing or refraining from doing, and it constitutes whether some behavior is an action (ibid.). If an agent could not have done otherwise, what she did was not an action. The question of what it means to have the ability to do otherwise, to do something or refrain from doing it, is even intact in cases that put free will under pressure, as Pickard (2015) argues in the context of substance abuse and addiction. The addict is someone whose ability to do otherwise remains intact. The addict’s actions of consuming and using substances is something she can refrain from, and thus, the addict is *not* compelled to use drugs, although the addict’s capacity to do otherwise is weakened. She is *not* an unfree agent. For Pickard:

Our common sense conception of agency draws a basic distinction between actions and mere bodily movements, such as automatic reflexes. What makes a piece of behavior an action, as opposed to a mere bodily movement, is that it is voluntary, where this means that the agent can exercise choice and at least a degree of control over the behavior. […] [O]ne is responsible for actions, as opposed to automatic reflexes, because it is up to one whether and how one acts. (Pickard, 2011, 212).

The consequence of Pickard’s view is that we can hold the addict and other agents suffering from “disorders of agency” (Pickard, 2015) responsible for their doings because they are not compelled; they can and could have done otherwise. This means that even in cases where agency seems diminished and weakened, we still hold an agent responsible because she can do and could have done otherwise. Instead of blaming her, we hold her responsible (ibid., pp. 140–2).

For theories such as the ones just mentioned, the greeting example is an action of mine for which I am responsible, even though I am not conscious of it in a strong sense that requires knowledge of the intention with which it was done. Rather, I am responsible because we have expanded the field of action such that it is broader than the scope of reflective consciousness. This expansion allows us to keep action coextensive with responsibility.

As for the logical possibility where action and consciousness are kept coextensive, but it is denied that action and responsibility are so, we would have to imagine a theory that denies our intuitions about which behavior we should feel responsible for. Here, the attempt is to argue that there is no problem with thinking we are responsible in the greeting example despite the fact that the way of greeting is not part of my action. This solution would thus preserve the tie between action and consciousness but at the cost of sacrificing our intuitions about which behavior we should feel responsible for.

What we see is that we can either (1) keep both assumptions synchronically, but then the greeting case must be denied. We can (2) deny that reflective consciousness is coextensive with action and accept that the scope of action must be broadened in order to keep the link between action and responsibility, or (3) we can deny that action and responsibility are coextensive and argue that we can be responsible for behavior that is not action. Thus, either we cannot explain the greeting case, or we are forced to give up at least one of the intuitive assumptions often thought central to action theory.

I wish to propose a fourth option that preserves both assumptions mentioned above. My aim is to do justice to our intuitions about which behavior we should feel responsible for. The crucial step of my argument is to deny an underlying assumption that has been governing all sides of the debate so far. This is the assumption that when we evaluate the status of some behavior to determine whether it is an action, then it is solely the contemporaneous consciousness of the individual we need to examine. Instead, I want to argue that only as far as I *can* become conscious of some description under which the action was intentional does my behavior deserve to be called an action. This account entails that some actions of mine can return to me as questions, to which I can only appropriate the reply diachronically, over time.

In the discussed logical strategies (1)–(3), we find what I will call the synchronicity assumption to be operative: The states of consciousness relevant to determining whether some behavior is an action are only those states that are synchronic or contemporary with the behavior. Synchronicity refers to the theoretical role of immediate first-personal insight into the intention of one’s action (see also Ingerslev, 2020). There is a tendency to identify as a hallmark for agency the simultaneous relation between one’s first personal insight into one’s intention and the action being performed. That is, if consciousness of intention is relevant in order to classify whether something is an action or not, then it is synchronically relevant. However, we do not always have immediate insight into the intention of our habitual doings and, in some cases, can only gain this insight over time.

Either it is required that consciousness of the intention is decisive for whether something is an action. Therefore, habitual doings where one lacks insight into one’s intention are not actions, or habitual doings are considered actions, but then knowledge of one’s intention is not required for something to be an action. Practically, this means that upon asking an agent what she is doing, either she would immediately know the intention with which she is doing something or she would understand more broadly which practical activity she is engaged in, although she is performing it unthinkingly. Thereby, the possible awareness of habitual actions is synchronically related to the possibility for self-understanding. In this way, full self-understanding afforded by an agent’s awareness of her habitual behavior is immediately available by the time of the awareness. The general tendency expressed by the synchronicity assumption is at play in all the positions presented provided above.

In the case of unreflective manners of greeting, we can thus either argue (1) that it is not part of my action because, synchronically, I do not know of an intention that would make this behavior intentional; (2) unreflectively greeting in this particular way is an action of mine because, synchronically, I am embedded and enactively involved as agent also in unreflective cases. I could, synchronically, have refrained from greeting this or that way; I was not compelled. (3) Synchronically, I am unaware of my doing, but I remain responsible as the action is ascribed to me at the time T.

By contrast, I wish to argue that it is by a process of appropriation of our intentions that we are able to understand some of our unreflective doings as actions. The description under which my action is intentional is still relevant to determine whether some behavior of mine is an action. However, it is not the synchronically accessible description but rather those aspects that I *can at some time T* become conscious of as belonging to a description under which my action was done intentionally. Diachronically, therefore, I can assume ownership of my doing in such a way that I understand my actions retrospectively as belonging to me in a stronger sense. I take on the responsibility of having done them for a reason. I have been responsible all along, but I come to assume ownership and responsibility for my action over time. Rather than my greeting behavior being an accidental aspect of my action, I can appropriate it as something I did; I have come to realize that I do greet my colleagues differently for a reason. Maybe, without having thought about it explicitly, I do think that my one colleague prefers a serious work ethos and therefore I greet him in a more formal way. What characterizes behavior of mine that I can come to appropriate over time as an action of mine is that this behavior can come to matter rationally for my self-understanding. Instead of providing retrospectively a causal explanation of something I did, I provide a rational account that matters for how I see myself as a person. If asked why I keep being distant and snappy at my good friend, I might come to realize that I have been angry with her for a long time. If I believe that due to stress and a heavy workload I could not have acted differently, I would have explained my aggressive tone causally. By contrast, appropriating my actions diachronically means to engage with my behavior and try to realize for how long I have acted like this and, in responding to these doings, to understand them as something done by me for a reason. This might lead to doubts whether responsibility can be thus construed retrospectively and to the objection that forms of antirealism concerning action will be unavoidable. I will return to these issues in Section “*Objection: Does Diachronicity exclude first-personal authority?*”

Repetitive behavior of a certain opaque and incomprehensible kind is at the same time something I can come to understand as actions of mine. Whether I keep smoking, keep greeting my friend seriously, or whether I might have been in love with someone for a long time without realizing it, these cases entail behavior that I can diachronically come to realize that I did for a reason. Therefore, I can appropriate them as actions of mine and take responsibility for them. Whereas the cases and consequences will differ between smoking out of habit, greeting my colleagues differently, bullying or discriminating against someone, the structure of how we diachronically assume responsibility for our past behavior is the same. What I aim to show in this paper is that there is such a thing as appropriating one’s past behavior as action.

By realizing that the scope of conscious action for which I am responsible also encompasses cases where the consciousness of action occurs diachronically, we can account for how it is that self-understanding is important *even* in cases of unreflective actions. If we accept synchronicity, many of our daily routines and other involuntary aspects of our unreflective doing are in danger of being out of our conscious reach. That is, a consequence of denying that we can diachronically appropriate behavior as actions of ours is that we are unable to account for how we increase our understanding of our past agency by reflecting on our reasons for earlier behavior. Without this diachronic option of appropriation, my self-understanding becomes opaque, blocked, or even barred. Ultimately, self-estrangement can be a result of not accepting and acknowledging diachronicity as a process of appropriating one’s own habitual doings as actions.

The field that opens with diachronicity is larger than what I aim to address in this paper. My aim is to focus on cases where part of my emotional response to certain situations is beyond the synchronically available description that makes my action intentional. However, with a diachronic perspective, some of my habitual doings can be appropriated, and what was blocking or barring an adequate account of my self-understanding is opened up. In what follows, I will look into a case of inhibited intentionality and propose that we understand it as a case of weak agency. This will help us address the challenge for action theory, namely, the status of unreflective action as personal.

## Inhibited Intentionality

In this section, I will argue that those unreflective actions that matter for our self-understanding and can be appropriated diachronically are not merely of a peripheral kind, such as the greeting case. Many of our daily patterns of behavior, for example, those that are socially and culturally shaped, fall within the scope of unreflective action.

Many involuntary aspects are automatic and can play no role for my self-understanding; that is, they do not fall under the scope of what is personal. Consider how many times I blink per minute or the size of personal space measured in relation to how I place objects belonging to me when traveling by train. These are aspects of my actions that I do involuntarily and with little sense of agency involved in them. However, some involuntary aspects of my actions do play an important role for my potential self-understanding as an agent. Maybe I have to look down when talking to certain persons, maybe I cannot use my body normally when I throw a ball, or maybe I cannot sit far away from my personal belongings and have to cling to my purse in order to feel safe in a public train. These involuntary aspects are expressed in my bodily habits. In order for me to understand them as belonging to me in a stronger sense, that is, as something I do and that I am responsible for doing, the diminished sense of agency at stake in these forms of action must be addressed. Such doings of mine play an important role for my self-understanding. If I keep avoiding certain persons, if I feel unsafe without my personal belongings clinging to me, or if I greet my friends differently, I must know why I do so in order to understand my own actions as mine and to understand who I am as a person.

In what follows, I will take up Iris M. Young’s example of throwing a ball in a certain way. In her example (Young, 1980), she addressed the inhibiting effect that cultural education can have on a person or a group of persons and their possibility for self-expression and self-understanding. In the case of throwing like a girl, she makes the philosophical point that embodied intentionality can be inhibited by cultural upbringing. Whereas this is less surprising, I will focus on her theoretical explanation of how such inhibition is experienced. I am interested in the example in relation to the question of how the involuntary aspects of our habitual doings can still be appropriated as something done by us, which makes inhibited intentionality a case of weak agency.^4^

In Iris Young’s 1980 paper, she proposes that embodied intentionality can be experienced and enacted as inhibited. Young discusses the role of gender by questioning how we embody intentionality. She claims that—in a specific culture, at a specific time in history—throwing, standing, walking, talking, sitting, and laughing is experienced differently relative to one’s gender. Young does not make an exhaustive claim, but her point is that the ingraining and bodily habituation of gender stereotypes through various social processes come with forms of inhibited intentionality for women. In order to assess this claim, we need to make certain preliminary considerations. For Husserl, the notion of bodily awareness is characterized by an experiential “I can.” This “I can” neither is a belief nor is it experienced consciously as a propositional truth. Rather, embodiment is enacted and prereflectively experienced under the condition of an “I can,” understood as practical possibility (Husserl, 1989, p. 159 ff., p. 165 ff., p. 269 ff.). Another way to phrase this is that the way we experience embodiment prereflectively is as the capacity to do…. Or the ability to…. Being embodied means to be intentionally directed toward the world and to feel moved by the world, i.e., by objects, people, and situations. Movement, action, and activity are thus structurally characterized by an embodied ability to…. Further, this is a way in which we embody consciousness: The prereflective awareness of “I can” is constitutive of how we embody intentionality; we are directed toward the world as embodied beings. As we said above with Merleau-Ponty, the affordance character of objects, situations, and other people is perceived through the lived body, and the responsive answering of these calls is experienced as an embodied capacity, the “I can” as an experiential structure that shapes our bodily existence. Practical understanding is an embodied practice that precedes theoretical knowledge and propositional attitudes. We inhabit a world practically before we understand it theoretically. In this way, embodied intentionality is operative beneath our consciously minded actions. To put it differently, the experiential structure of embodiment is an “I can” that mediates our bodily movements and our comportment and is prior to representational, theoretical understanding.

Young investigates the idea that women comport themselves differently from men and illustrates this by the example of throwing a ball:

Women tend not to move out and meet the motion of the ball, but rather tend to stay in one place and react to the ball’s motion only when it has arrived within the space where she is. The timidity, immobility and uncertainty which frequently characterize feminine movement project a limited space for the feminine “I can.” (Young, 1980, p. 150).

Young argues that there is something like feminine bodily existence where the embodied intentionality is experienced as inhibited in the sense of an “I cannot.” The idea is that the “I can” remains fundamental for our embodied existence but that it can be modified as we take on certain cultural life forms. Space is culturally shaped and coded such that certain groups are allowed to move in certain ways as they follow the forms and norms of collective education.

What Young refers to as feminine bodily existence neither is meant to be exhaustive nor is it meant to be universal (ibid., p.139). Her account “claims only to describe the modalities of feminine bodily existence for women situated in contemporary, advanced industrial, urban, and commercial societies” (Ibid., pp. 139–40). That is, she specifically targets a kind of comportment that is set in time and space, which she terms feminine bodily existence, and she seeks to describe its phenomenological structure.^5^ I believe the strength of this account lies less in how it describes feminine bodily existence and more in how it sheds light on the possibility that agency can be weakened despite the fact that the agent is free. With Young’s example, it is possible to expand the field of possible involuntary aspects of one’s doings from our local greeting example to the more global case of cultural life forms under which certain groups suffer. I believe that Young did not develop the potential of her account in the broader field of philosophy of action. The notion of inhibited intentionality is fruitful for our understanding of the relation between involuntary aspects of agency and the possibilities for self-understanding.

For a subject that experiences feminine bodily existence, embodied intentionality is inhibited. According to Merleau-Ponty, the lived body structurally describes how subjects are embedded in and belonging to the world. To embody an “I can” means to be intentionally directed toward this world by being capable of…. Normally, the lived body is the unifying synthesis of our experiences. For Merleau-Ponty, a bodily synthesis refers to how the lived body ensures and enables the unity of embodied perception. When I see a cup, I experience the synthesis of my perceptual impressions, not through my mental representation of a cup, but because of the primordial rootedness of perceptual affordances in my lived body. My perceptual act is an embodied comprehension of the cup:

[T]o habituate oneself to a hat, an automobile, or a cane is to take up residence in them, or inversely, to make them participate within the voluminosity of one’s own body. Habit expresses the power we have of dilating our being in the world, or of altering our existence through incorporating new instruments. (Merleau-Ponty, 2012, pp. 144–5).

This means that “[c]onsciouness is being toward the thing through the intermediary of the body” (ibid., p. 140). Here, consciousness is embodied in such a way that every perceptual act is rooted in bodily practical understanding. Thus, what is embodied in practical understanding is at the same time an incorporation of one’s world. As Merleau-Ponty phrases it: “A movement is learned when the body has understood it, that is, when it has incorporated it into its ‘world,’ and to move one’s body is to aim at the things through it, or to allow one’s body to respond to their solicitation, which is exerted upon the body without any representation” (Ibid., p. 140). The body is not “in” time or “in” space, but inhabits times and space as an active linking them together. Thus, “[i]nsofar as I have a body and insofar as I act in the world through it, space and time are not for me a mere summation of juxtaposed points, and no more are they, for that matter, an infinity of relations synthesized by my consciousness in which my body would be impacted” (Ibid., p. 141). The synthesis of the body is thus that of having a world, or “understanding its world without having to go through ‘representations,’ or without being subordinated to a ‘symbolic’ or ‘objectifying function”’ (Ibid., p. 141). In the case of feminine bodily existence, the embodied synthesis is disrupted. For someone who exists in this feminine way, the unifying synthesis provided by the lived body is blocked; thus, the experiencing subject remains stuck in not expressing herself fully and not receiving her world fully. She is detached in some aspects from worldly belonging. She does not manage full worldly transcendence, and she experiences herself—through her embodied existence—as someone who feels less capable, less open, less powerful, more insecure, and more concerned with her own fragility (Young, 1980, pp. 143–4); she is held back in immanence (ibid., pp. 144–5). The world for a woman is constituted as a field of inhibition rather than as a world to be inhabited. Further, she experiences embodiment as a something that is not tied to fields of possibilities because these possibilities in fact occur as possible for someone else than her; she is caught in the lived absence of possibilities and thus in lived inhibition. Experientially, the field that opens with an embodied “I can” is closed off for her, and as a field of inhibition, it does not offer her the means of self-expression; she experiences this field as indicative of her own incapacity, i.e., her embodied intentionality is experienced as inhibited (ibid., p. 147).

Typically, the feminine body underuses its real capacity, both as the potentiality of its physical size and strength and as the real skills and coordination which are available to it. Feminine bodily existence is an *inhibited intentionality*, which simultaneously reaches toward a projected end with an “I can” and withholds its full bodily commitment to that end in a self-imposed “I cannot.” (Ibid., p. 146).

Granted that we are all limited in our bodily capacities, the experienced “I cannot” belongs just as much to the nature of embodied intentionality (Ingerslev, 2013). The case Young is making is that for feminine bodily existence, the experienced “I cannot” is self-imposed: “When the woman enters a task with inhibited intentionality, she projects the possibilities of that task—thus projects an ‘I *can*’—but projects them merely as the possibilities of ‘someone,’ and not truly *her* possibilities—and thus projects an ‘I cannot”’ (Young, 1980, p. 147).

This point is crucial for our present task of arguing that inhibited intentionality is an aspect of weak agency that can be appropriated diachronically. The experience of a self-imposed “I cannot” shows an aspect of who I am and what I do that might at the moment be something that I am not conscious of. I do not know that I inhibit myself, but it remains something for which I am responsible and that I can come to realize—maybe upon rumination, critical thinking or therapy—as something I did to myself. This is where the notion of diachronic self-understanding comes into play. It is not a question of taking more sports classes in order to come to throw more fully. Rather, it is a matter of appropriating an embodied worldview, a practical self-understanding, as something I have acted under and that I might want to change. If we could not diachronically come to realize our own reasons for action, then our theory of action cannot explain why today, upon acquiring this consciousness, I should feel responsible, or importantly characterized as an agent, by my own past self-inhibition.

Young’s claim is that feminine bodily existence isolates movements and does not make use of the full bodily potential to perform an activity. As illustrated by the example of throwing, a woman might only use her arm in throwing a ball. By comparison, a non-feminine bodily existence would turn the upper body, use the strength of a firm grounded position, and the other arm in aiming, etc.: “The undirectedness and wasted motion which is often an aspect of feminine engagement in a task also manifests this lack of body unity” (ibid., p.147). Not only is there an undirectedness or a waste of motion in the sense that some movements could be more focused and fully executed, but this is the superficial part of the problem. The real problem is the lack of bodily unity. In reaching for the objects, grasping, moving, and perceiving, the female body is only practically set in motion; a woman does not carry her movements fully through. Thus, no bodily synthesis can be fully made. This means, in its widest consequence, that the bodily synthesis is disrupted and that forms of self-estrangement and derealization are part of feminine self-understanding. To see why that is, we must return to the synthesis of the lived body, as Merleau-Ponty understands it. As said above, the embodied synthesis anchors my experiences, and it provides the background for my self-understanding altogether:

[T]he consciousness that I have of it [my body] is not a thought, that is, I cannot decompose and recompose this consciousness in order to form a clear idea. Its unity is implicit and confused. It is always something other than what it is: always sexuality and at the same time as freedom, always rooted in nature at the very moment when it is transformed by culture, it is never self-enclosed but never transcended. […] Thus, I am my body, at least to the extent that I have an acquisition, and reciprocally my body is something like a natural subject, or a provisional sketch of my total being. The experience of one’s own body, then, is opposed to the reflective movement that disentangles the object from the subject and the subject from the object, and that only gives us thought about the body or the body as an idea, and not the experience of the body or the body in reality. (Merleau-Ponty, 2012, p. 205).

When the bodily synthesis is disrupted and self-understanding is realized as derealization and self-estrangement, the experiential field itself is experienced as inhibiting, threatening, and possibly even foreign. What could have been part of a world-for-me is turned into a foreign field that alienates me from myself. As a result, feminine bodily existence is discontinuously realized: “feminine bodily existence stands in discontinuous unity with itself and its surroundings” (Young, 1980, p. 147). Overall, feminine bodily comportment is characterized as the failure to make full use of the body’s spatial and lateral potentialities (ibid., p. 142). When I experience myself as bodily inhibited, no bodily synthesis can be fully reached. As a result, my self-understanding is equally shattered and disrupted since I cannot understand why I feel and behave this way. The relation between my movements and my world, my movement and reality, is in this way disrupted and inhibited.

## Inhibited Intentionality as an Expression of Weak Agency

In this section, I will introduce the notion of weak agency with the aim of specifying what is characteristic for unreflective and habitual behavior that can be appropriated as actions of mine. If we take up the idea of feminine bodily existence as described by Young, it is a technical term for how I act under internalized superimposed structures. The way I throw, walk, or address people differently is part of my unreflective actions. I might not be consciously aware of how I walk, and while strangely unconscious of my gait, it remains my way of walking. This aspect of my bodily habits calls my agency into question (Ingerslev, 2020). If I am not aware, say, of how I cringe in front of certain authorities (Freud, 1914; Lear, 1998), how can I assume ownership of these doings of mine as actions? If I am acting out fearful and inhibited behavior or emotional traumas (Freud, 1909), how do I come to appropriate these actions as mine; how do I take responsibility for them? What is special about the involuntary aspects of inhibited intentionality is that they are tied to our self-understanding while being temporally beyond our control. This means that something I do that involves inhibiting my field of action is at the same time crucial for my possibility for self-understanding. If I am the one throwing the ball in an inhibited way, or cringing in front of authoritative persons, I must know how these doings belong to me in a stronger sense. The self-imposed inhibition entails an inkling question: how do I appropriate my doings as actual actions of mine?

We might live a whole life without knowing about the depth of cultural influence on our behavioral pattern. We might remain ignorant of the many layers of body memory that affect our ways of responding to people and situations (Fuchs, 2012, 2018). However, what the notion of weak agency allows us to account for is the coincidence of personal habits with lived forms of self-estrangement that are even at times self-imposed. If we take Young’s insights further, the idea is that these involuntary aspects of habits can shape a life form that disrupts one’s self-understanding and makes it difficult to endorse one’s actions as one’s own; the latter remain foreign in nature to the agent, and as a result, the agent’s self-understanding becomes distorted.

The technical term *Weak Agency* aims at specifying forms of unreflective and habitual doings that can be appropriated over time as one’s own. By reference to Young’s claim that intentionality can be inhibited and, further, can be taken on as a self-estranged life form, the notion of weak agency pursues the possibilities of self-understanding within forms of behavior that otherwise seem less agential or less free. Limit cases of weak agency where hardly any agential freedom is at stake can be found in life forms where the self-estrangement and the objectification is close to total. Such cases entail less of an opening for transformation and appropriation over time (Honneth, 2008). When agency is no longer simply weak but blocked, a certain life form is destructive and cannot be appropriated as in the commodification of bodies or dehumanizing reification of human lives mentioned by Honneth. We can think of cases of hierarchical or religious indoctrination. In such cases, the only diachronic understanding of my past behavior available is that someone else made me think or act in a certain way. I cannot come to appropriate that I had a reason for behaving the way I did. For whatever reason there was for my behavior, it was not a reason of mine but of those who indoctrinated me. Habitual behavior differs from these limit cases in that we find within the habits themselves an opening for appropriation of one’s own behavior. The openness of one’s own habits to transformation is tied to our possibility for self-understanding. Diachronically, I can come to take responsibility for the inhibited intentionality that shapes my unreflective doings, and thereby, I can come to appropriate them as actions of mine. Iris Young’s case of feminine existence is thus an example of a life form that can lead to self-estrangement, but it also entails an opening for appropriation; it is a case of weak agency.

The reason why involuntary structures are interesting for our understanding of weak agency is due to the tension between a diminished sense of control and the intimate familiarity in bodily habits. We want to understand how the involuntary aspects of habit are more than just impersonal happenings, as they belong to me in a strong sense; I am the person who is acting freely, yet I am involuntarily inhibited in my bodily existence. I am the one acting under superimposed internalized structures, yet I am also the one who can take responsibility for this doing diachronically and thereby come to understand something about myself. The notion of weak agency thus captures that the degree to which the agent experiences herself as the author of her own actions can differ, i.e., the degree with which an agent feels in charge of her own actions can vary. When this degree is low, the agent might not synchronically sense herself as the author of her actions in a strong sense. She might know explicitly that she is doing something or that she often does this or that, but she might not intend to do so or want to do so. The weakness at stake can thus be characterized as unwilled, as inattentive or unfocused doings, or even as an involuntary act, not because the action is forced but because it occurs independently of the agent’s contemporaneous self-conscious understanding of her actions. By contrast, strong agency is tied to cases where we deliberatively and with reason perform certain kinds of actions; we consciously authorize a certain doing, and we are capable of providing the reasons of our action. I decided to take the job, or I realized our friendship could not continue; in these cases, a process of deliberation leads to a decision and culminates in action. In taking the job or ending a friendship, the agent senses agency as authorship in a strong sense; this is a doing of mine. It is not difficult to see how such doings play a central role for our self-understanding. I want to be the kind of person who is a good friend in this particular way; this is not possible with Y, and thus, I have to end the friendship. Integrity is one way to describe such a relation (Korsgaard, 1996; Crowell, 2013). A strong agent is someone who, upon consideration, provides reasons for her action: Do I want to go out for a beer and be a good colleague, or, do I want to pick up my son and take him to football training (Crowell, 2013)? The measurement for my integrity is the normative source of my actions. I take responsibility for wanting to be a good colleague, prioritizing collegial chat over a rainy day at the football field.

What is weaker in the cases I am interested in is the kind of self-understanding of what one is doing that is available to the agent presumably acting. In cases of weak agency, an immediate response to the question why are you doing X is not available to the agent. However, the response can be appropriated diachronically. Weak agency is a term that covers the remaining possibility for appropriating the full scope of one’s actions as one’s own. Weak agency differs from ignorance in that I can come to be aware of cringing and throwing like a girl, but I cannot immediately change it. Ignorance would be the case, where the scope of one’s actions relies on epistemic barriers, not on inhibited or blocked body memory. If someone tells me that my favorite chocolate brand is run by an evil company that exploits children and women in the third world, I will stop buying it. If someone tells me, I throw like a girl, I would have to appropriate my whole being over and over again while committing to the field of my possibilities given my history while at the same time becoming the person I am. This means that the freedom involved in cases of weak agency is confined. Whether I throw like a girl or not, whether I address my embodied inhibition in therapy, these are ways in which I embody a confined freedom; I commit myself with the sedimented bodily history I have to become the person that I am—over and over again.

Merleau-Ponty reflects on this kind of confined freedom and its relation to a commitment to self-understanding by referring to the therapeutic relation in psychoanalysis. The example serves the purpose of illustrating what is meant by diachronic appropriation characteristic of weak agency:

*By taking up a present, I* again *take hold of my past and I transform it, I alter its sense, I free myself and detach myself from it. But I only do so by committing myself elsewhere. Psychoanalytic treatment does not heal by provoking an insight into the past, but by first relating the subject to his doctor through new existential relations. “Merleau-Ponty, 2012, p. 482 (Emphasis added by the author)”].*

…[I]t is a question of re-living the past as signifying this or that, and the patient only achieves this by seeing his past from the perspective of his coexistence with the doctor. The complex is not dissolved by a freedom without instruments, but rather is dislocated by a new pulsation of time that has its supports and its motives. The same is true for all moments of insight: they are actual if they are sustained by a new commitment. (Ibid., p. 482).

The notion of commitment at stake here is one that refers to possible self-understanding rather than that of a onetime promise or resolute decision. It refers to what Lear calls a dreamlike engagement, where the proper meaning of some behavior is not fixed and not identical to the manifested one (Lear, 1998 p. 97 ff.; Lear, 2017, p. 102, see also Merleau-Ponty, 2006, pp. 177–179). It is not the case that I once and for all *decide* to not throw as a girl or not cringe in front of authorities, but I commit to appropriating this behavior of mine, which will take the shape of recommitment, something I will have to do over and over again. The process of appropriation might involve several attempts to make sense of various happenings over time, cringing, not cringing, being fearful, trying not to be, etc. The commitment over time to the quest for self-understanding might at some point allow me to not cringe. Even if I keep cringing despite myself, I might work with these involuntary aspects of my actions as part of my appropriation and conscious quest for self-understanding. Commitment means that I aim for self-understanding and strive to gain insight into my reasons for acting; only the description under which my action is intentional might not always be synchronically accessible to me.

The appropriation of one’s unreflective doings thus differs from being resolute, making up one’s mind or having enough will power to change one’s habitual behavior. Rather, it consists in the attempt of coming to terms with weaker forms of agency for which I nonetheless take responsibility. This is why Merleau-Ponty’s description of confined freedom is tied to the example of commitment in psychoanalysis. In therapy, the agent addresses the involuntary aspects of her embodied personal history in order to commit herself anew as a free but weak agent. We can understand this kind of commitment as a diachronic appropriation of one’s past doings *as* actions for which one take responsibility by taking them up as *part of* one’s history. Thereby, the agent commits herself to striving for self-understanding. She comes to rediscover her past as a possibility for future self-understanding:

*Freedom lies in the re*discovery *of my habitual past as a* reservoir *of possibilities, indeed, as a vigorous force actively shaping my future at every moment. It lies in our ability to enter into this force, both past and futural, intrinsically rigid and intrinsically flexible, with the stance of one who approaches the world as a place where meaning grows. “Talero, 2006, p. 203 (Emphasis added by the author)”.*

What Talero describes as intrinsically rigid and at the same time intrinsically flexible is the nature of our bodily habits and unreflective actions. The involuntary aspects of these doings remain a part of the field that actively shapes our future, and as such, it can be appropriated (Ingerslev, 2020), however, diachronically, over time, as Merleau-Ponty describes. The process of approaching the involuntary aspects of one’s doings and projecting them toward the world is the process of striving for self-understanding in cases of weak agency. That freedom is confined means that it does not exist outside a historical past but that this past must be appropriated over and over again.

Importantly, appropriation is not similar to being concerned with finding a set of lost intentions that we then take on, through an external third-person view on our own lives that conveys a certain useful meaning. I will return to this point in Section “*Objection: Does Diachronicity Exclude First-Personal Authority?*.” It is not the case that I rediscover in my past aggressive behavior that I was indeed upset and angry with my good friend, and that is it. As Merleau-Ponty argues, it is a matter of an existential commitment that unfolds over time. While emphasizing how the involuntary aspects of our habitual doings can be diachronically appropriated, the point is not that we can simply attribute an intention to our former behavior, as if rewriting our personal history. Rather, the structures of body memory, inhibited intentionality, and emotional trauma entail an openness that returns to us as a question for our self-understanding: Why am I doing this, or why did I do it again? This opening is constitutive of weak agency, and it enables me to commit again to making sense of what I am doing as a person. The worry expressed here concerns the processual aspect of appropriation. Whereas realizing that I was upset with my good friend provides me with a reason for my past behavior, the attempt to take responsibility for my actions might entail several attempts at appropriation and at accepting and endorsing my weak agency. This is the difference spelled out between an existential commitment and the attempt of finding a reason. The former is an ongoing process that defines me as a person that actively engages with my personal history. The latter could be a case of ascriptivism, where I am unconstrained by past affairs in which intentions I ascribe to myself to explain my action. The example of a therapeutic relation is helpful in order to illustrate how we will not come out as strong agents and how we will not get rid of all of our habits. Rather, we might learn why we repeat certain patterns of behavior. The therapeutic questioning of one’s behavior might help us to gain a richer self-understanding. It might help us to appropriate our past behavior as something we did intentionally and something for which we are responsible. Weak agency involves repetitive patterns of behavior that we will not as such get rid of simply by finding the reason why we repeat them, but what we do gain is insight into our own weak agency. Weak agency thus entails an important possibility for self-understanding; however, in order to see this, we needed to spell out the diachronic relation between action, consciousness, and responsibility in unreflective actions and in cases of inhibited intentionality.

## Objection: Does Diachronicity Exclude First-Personal Authority?

Obviously, much remains to be explained if we accept the notions of weak agency and diachronic appropriation. What are the temporal limits to what can be appropriated; what are the epistemological constraints on what can be appropriated; what is the interrelation between memory and self-understanding over time? Does diachronicity imply antirealism about actions, that is, can any past behavior of mine be appropriated over time as an action of mine? In what remains of the paper, I will briefly discuss whether the case of diachronic accounting implies seeing oneself from a third person-perspective and whether it implies the possibility of freely ascribing intentions *post hoc* to past behavior in order for it to take on the shape of action. One possible objection is that non-observational first-person authority is needed for an agent with respect to her actions in order for her to be a rational agent in the first place. It seems that diachronic accounting consists in an external perspective on oneself and would thus exclude self-understanding or uphold possible self-estrangement or inauthenticity. Therefore, it seems that accepting diachronicity and weak agency leads to a constructivist self-interpretation where we unbind ourselves from our own past in accepting third personal theoretical descriptions of our behavior that we could not immediately and rationally endorse at the time of action. These descriptions are instead theoretically construed by taking an external perspective on ourselves.

Richard Moran thus argues that the therapeutic relation could contaminate first-personal authority:

In various familiar therapeutic contexts, for instance, the manner in which the analysand becomes aware of various of her beliefs and other attitudes does not necessarily conform to the Transparency Condition. The person who feels anger at the dead parent for having abandoned her, or who feels betrayed or deprived of something by another child, may only know of this attitude through the eliciting and interpreting of evidence of various kinds. She might become thoroughly convinced both from the constructions of the analyst, as well as from her own appreciation of the evidence, that this attitude must indeed be attributed to her. And yet, at the same time, when she reflects on the world-directed question itself, whether she has indeed been betrayed by this person, she may find that the answer is no or can’t be settled one way or the other. So, transparency fails because she cannot learn of this attitude of hers by reflection on the object of that attitude. She can only learn of it in a fully theoretical manner, taking an empirical stance toward herself as a particular psychological subject.” (Moran, 2001, p. 85).

According to Moran, the difference between a theoretically formed perspective on oneself and a practical endorsement of one’s attitude toward oneself remains even when therapy seem to have unearthed a historical truth for a person:

*The person might be* told *of her feelings of betrayal, and she may not doubt this. But without her capacity to endorse or withhold endorsement from that attitude, and without the exercise of that capacity making a difference to what she feels, this information may as well be about some other person or about the voices in her head. From within a purely attributional awareness of herself, she is no more in a position to* speak for *her feelings than she was before, for she admits no authority over them. It is because her awareness of her sense of betrayal is detached from her sense of the reasons, if any, supporting it that she cannot become aware of it by reflecting on that very person, the one by whom she feels betrayed. The rationality of her response requires that she be in a position to* avow *her attitude toward him, and not just describe or report on it [.] (ibid., p. 93).*

For Moran, two features are central for self-understanding and thus for rational agency, namely, immediacy and transparency. Immediacy refers to the non-observational status of my self-knowledge. I immediately, without observation, evidence or inference know what my beliefs are, what I am doing, what I think. That is, immediacy is the epistemically privileged position I have toward my own mental states compared to the access of others to my mental states; they have to rely on observation and evidence-based reports to know something about my mental states (ibid., p. 92). For Moran, transparency is a condition on self-knowledge of beliefs that obtains when I determine which beliefs I have by reflecting on the worldly matter they concern. Transparency fails when the way I determine my beliefs is by reflecting on my own mental states or by observing my own behavior. Avowal of one’s beliefs occurs when, as a result, of reflecting on the state of the world with which my beliefs are concerned, I come to endorse the beliefs in question.

We can see how transparency would fail in the case of weak agency and thus how the possibility for self-understanding would be excluded.^6^ Moran describes the failure of transparency in the following way:

*[F]or our analysand, if she is unable to learn of her attitude toward the person by whom she feels betrayed by thinking about* him, *if here she can only attribute beliefs to herself but cannot avow them, then she will not come to avow them by engaging in more and better attributions to herself. (The theoretical stance toward oneself constitutes itself as self-sufficient realm.) When I deliberate about something, the conclusion of my deliberation settles the question for me only in virtue of my attitude toward this activity, not in virtue of what I may belief about its effect on me. The aim and conclusion is the binding of oneself to a certain course of action (or proposition), not the production of a state of mind that I might then treat as (further) empirical evidence of how I should proceed.” (Ibid., p. 95).*

Avowal, according to Moran, is the attitude with which I endorse the beliefs I have and when I make my first-person reports without any reference to evidence or inference. It is how I make up my mind and decide which beliefs of mine I endorse as true. The ability to avow my beliefs is constitutive my behavior as a rational agent: “A belief that cannot be avowed is thus cognitively isolated, unavailable to the normal processes of review and revision that constitute the rational health of belief and other attitudes. Thus we could explain why it is that the capacity not just for awareness of one’s belief, but specifically awareness through avowal, is both the normal condition and part of the rational well-being of a person” (Ibid., p. 108). The difference between making up one’s mind and “gesturing one’s mind” (ibid., p.122), as Moran characterizes the analysand’s verbal reports, is that in the first case, the agent responsibly and actively endorse her beliefs. Lying on the couch, verbally gesturing one’s mind, one is passively dissociated from one’s beliefs; one observes, finds evidence in one’s own reports, or discovers in the reports—together with the analyst—something about oneself (ibid., p. 114 ff.) To both the analyst and oneself, such reports occur as data, as “more (verbal) behavior for interpretation” (ibid., p. 121). The agent does not speak her mind, she reports something she does not first personally avow, and her reports are treated as data, as indications of something else both by the analysand and the analyst, according to Moran.

Now, the point we find in Merleau-Ponty’s quote above that the therapeutic relation consists in an existential commitment does not imply that we take on an empirical stance toward ourselves. Rather, the point is that, as part of an ongoing process of self-understanding, non-trivially, we come to discover certain things about ourselves that we then attempt to appropriate as actions of ours. If we accept Moran’s account of self-knowledge, an objection to the notion of weak agency would be that any such agent would not have self-knowledge, as transparency would fail in the cases of weak agency since the agent would self-observe in order to speak and know her mind. To be clear, my use of the term self-understanding differs from Moran’s notion of self-knowledge in the following way. In the broadest sense, both self-understanding and self-knowledge refer to what it means to know something about oneself. In the narrow sense, self-knowledge refers to the immediate and transparent way in which I avow my beliefs. For Moran, self-knowledge involved the ability to “avow one’s state of mind and not merely to attribute it to oneself” (Moran, 2001, p. 100). It is tied to the Transparency Condition, according to which “a statement is made by consideration of the facts about X itself, and not by either an ‘inward glance’ or by observation of one’s own behavior” (ibid., p. 101). This is Moran’s technical sense of the term. In between the broad and the narrow sense, I use the term self-understanding to refer to the kind of understanding I have of myself over time, that is, when I come to realize something about myself. Whereas self-understanding can also be immediate, as when I find out that I have a stomach ache, it can also be diachronic as when I find out that I have been disengaged in a friendship over many years. The discovery I make does not exclude avowal; I can come to realize that I must end a friendship that I have been ending indirectly, and I take responsibility for my past intentional actions involved in being disengaged. As a disengaged friend, I did not immediately understand what I was doing, but diachronically, I appropriate the past doings as mine, and my self-understanding avows my former weak agency. My use of the term self-understanding is thus broader than Moran’s technical use of the term self-knowledge, which only applies in cases that also involve immediacy and transparency. It is broader in that involves the temporal aspects of appropriation and thus of non-synchronic avowal; self-understanding includes diachronicity. In this particular sense, the notion of self-understanding that I apply resonate with some concerns tied specifically to the temporal aspect of Moran’s notion of self-knowledge (see Lear, 2004; Webber, 2017).

In accepting something like weak agency, the claim is that avowal is possible over time and that one can learn to speak one’s mind *diachronically*. In cases of weak agency, the dichotomy of relating either actively or passively to one’s attitudes (Moran, 2001, p. 114) is not accepted; intentional action can be appropriated over time. I come to realize something about myself that I was not able to endorse or understand earlier, and thus, I take responsibility for something I did in the past, i.e., something that was not just an unreflective, passive behavior of mine. This is not a trivial reading of what coming to terms with being who one is means, nor is it a blatant antirealism that claims that, by diachronic appropriation, any past behavior could be turned into an action of mine. Rather, it means that we get to keep our initial action theoretic assumptions; only the first assumption must be reformulated as follows:

*(1^∗^) My behavior deserves the status of action, when the description under which my action is intentional is synchronically or diachronically accessible to me*.

In combination with the second assumption, we get the following: If I am responsible, then the description under which my action is intentional is accessible to me synchronically or diachronically. With this reformulation, it is emphasized that the possibility for appropriation is the requirement for something to be an action. It does not imply that everything can be turned into something for which I am responsible or that anything can be turned into an action of mine over time.

In finding oneself repeating certain patterns of behavior, my behavior can occur to me as questionable. The process of questioning can turn into a commitment not to repeat oneself. However, such a commitment is vulnerable and might have to be repeated itself. It also means that certain attempts at appropriation might lead to self-misunderstanding and thus will have to be revised, as they cannot be appropriated or endorsed after all [Freud (1938) is especially clear on this point]. Appropriation thus means that my current self-understanding is an ongoing existential commitment:

I take hold of my past and I transform it, I alter its sense, I free myself and I detach myself from it. But I only do so by committing myself elsewhere. Psychoanalytic treatment does not heal by provoking an insight into the past, but by first relating the subject to his doctor through new existential relations. It is not a question of giving a scientific approval to the psychoanalytic interpretation, nor of discovering a notional sense of the past; rather it is a question of re-living the past as signifying this or that, and the patient only achieves this by seeing his past from the perspective of his co-existence with the doctor. (Merleau-Ponty, 2012, p. 482).

This movement or this strategy importantly involves a second-person perspective of engagement, not a third-person theoretical stance toward oneself. One commits oneself to the future by transforming what one already was into what one is through a responsive exchange of questioning one’s past behavior. In this way, my past is not a set of fixed reasons, but some of aspects of my past behavior are open for questioning and thus for attempts at appropriation of past doings of mine in the form of intentional action. The self-understanding involved is not inauthentic in that the agent blindly accepts a third personal view about her past behavior; rather, it is a responsive exchange that leads one to realize something about oneself. Psychoanalysis is used by Merleau-Ponty to show that we are not radically free and that we come to understand ourselves through a process of commitment realized in the second person perspective, through an “I–Thou” relation that facilitates our relationship to the past that we are and that we can come to endorse diachronically. The therapeutic relation thus serves as an example of diachronic appropriation, as it displays the mode of questioning and existential commitment.

## Concluding Remarks

The paper began by showing that certain cases are problematic for a standard theory of action, namely, cases of unreflective action where I intuitively feel responsible. I argued that the two basic and intuitive assumptions concerning the interrelation between consciousness, action, and responsibility can be kept if we accept a diachronic perspective on responsibility in cases of unreflective actions. I showed that cases of unreflective actions are not just in the periphery of the field of actions but are in fact at stake in culturally restricted patterns of behavior as well. Iris Young’s notion of inhibited intentionality provided a case that allowed me to expand the scope of unreflective actions to embodied cultural life forms. I further argued that such cases are better understood as cases of weak agency. With the notion of weak agency, we can see how unreflective actions and habitual behavior are forms of action, as they can be diachronically appropriated. I used Merlau-Ponty’s example of self-understanding in psychoanalysis in order to show how in appropriating our past behavior as action, we do not just take on any third personal description of our past that might seem suitable, but we assume responsibility for our past in coming to terms with being who we are. Appropriation is thus a rediscovery of one’s reason for having acted in a certain way, and at the same time, appropriation is a process where one commits oneself to being and becoming who one is. Taking up a present by committing myself elsewhere is a different way of saying that I appropriate my past behavior anew by taking responsibility for who I am as a person. Finally, I proposed that self-understanding of this kind differs from immediate and transparent self-knowledge at stake in first-person endorsement of my actions and beliefs. I proposed a kind of self-understanding to be possible even in cases of weak agency; thus, I emphasized how the process of reason finding in diachronic appropriation is exemplified by the second-person responsive process of committing oneself to transformation as in the therapeutic context. The challenges for this position are many, but the main insight is that by reflecting on responsibility in cases of weak agency, we get a new approach to studying the role of consciousness as well as the possibility for self-understanding in unreflective actions.

## Data Availability Statement

The original contributions presented in the study are included in the article/supplementary material, further inquiries can be directed to the corresponding author.

## Author Contributions

The author confirms being the sole contributor of this work and has approved it for publication.

## Conflict of Interest

The authors declare that the research was conducted in the absence of any commercial or financial relationships that could be construed as a potential conflict of interest.

## Footnotes

1 There are of course many ways in which we are conscious of ourselves when acting. For example, I am sensorily conscious of how my body feels, perceptually conscious of my surroundings, and I am also typically reflectively conscious of what I am doing and why I am doing it. My aim in this paper is to investigate the role of the latter form of reflective consciousness as a constitutive requirement for action.

2 In Section “*Objection: Does Diachronicity Exclude First-Personal Authority?*,” I return to Moran’s conceptions of first-personal authority and avowal.

3 In what follows, I shall propose a fourth argumentative strategy that draws on aspects of the Merleau-Pontian phenomenology (see sections “*Inhibited Intentionality As an Expression of Weak Agency”* and “*Objection: Does Diachronicity Exclude First-Personal Authority?”*). I shall, however, emphasize the first-personal agential insight in temporally expanded agency and thus endorse Merleau-Ponty’s analysis of confined freedom while accepting the framework offered by action theory. If we endorse merely the expansion of the domain of action to also encompass unreflective doings, we cannot spell out the possible self-understanding of an agent of her own doings over time. I thus attempt to spell out the action theory needed for engaging with the temporal aspects of agency that Merleau-Ponty does not himself address in his use of a psychoanalytic example, see Section “*Objection: Does Diachronicity Exclude First-Personal Authority?*”

4 I will focus specifically on the action theoretical question that Young’s influential and highly debated paper raises. This means that I will leave out important discussions of Young’s own assumptions and of the implications of her work.

5 In what follows, I will refer to “feminine existence,” “she,” “her,” “woman,” and “womanhood,” etc. as does Young. That means, independently of whether or not one believes such a thing to ontologically exist or to fit the descriptions given by Young, the theoretical claim I am interested in is that a modification of the underlying “I can” that characterizes embodied intentionality is possible and that this modification is described by the term “feminine” or “woman”.

6 A comment on the difference between my use of the term self-understanding and Moran’s use of the term self-knowledge will follow below.
